# MRI Predictors for Improvement Without Any Intervention of Clinical Symptoms in Patients With Lumbar Disc Herniation, Questioning the True Need for Surgery

**DOI:** 10.1155/rrp/4954622

**Published:** 2026-01-05

**Authors:** Andrea Šprláková-Puková, Matej Straka, Tereza Habas, Adam Čellár, Marek Dostál, Tamara Barusová, Marek Sova, Soňa Kryštofová, Martin Smrčka

**Affiliations:** ^1^ Department of Radiology and Nuclear Medicine, University Hospital Brno, Brno, Czech Republic, fnbrno.cz; ^2^ Department of Radiology and Nuclear Medicine, Faculty of Medicine, Masaryk University, Brno, Czech Republic, muni.cz; ^3^ Department of Biophysics, Faculty of Medicine, Masaryk University, Brno, Czech Republic, muni.cz; ^4^ Institute of Biostatistics and Analyses Ltd., Brno, Czech Republic; ^5^ Department of Neurosurgery, University Hospital Brno, Brno, Czech Republic, fnbrno.cz; ^6^ Department of Neurosurgery, Faculty of Medicine, Masaryk University, Brno, Czech Republic, muni.cz

**Keywords:** conservative therapy, disc herniation, lumbar spine, MRI, spontaneous resorption

## Abstract

**Introduction:**

Spontaneous resorption of a herniated lumbar disc and disappearance of clinical symptoms without repair is a well‐known but not well‐studied phenomenon. This prospective study uses magnetic resonance images to search for predictors as to the possibility of spontaneous herniation resorption without any intervention and patients’ predisposition to benefit from conservative treatment.

**Materials and Methods:**

Of the 125 patients examined by magnetic resonance imaging, 22 had clinical symptoms that spontaneously (without any intervention) disappear. The spinal condition of each was classified using Fardon’s, Modic’s, and Pfirman’s classifications, and physical dimensions of the affected disc and herniated disc were measured. Inter‐ and intrareproducibility of this measurement were determined. Predictors for spontaneous disappearance of clinical symptoms were selected using multivariable logistic regression and receiver operating characteristic (ROC) analysis.

**Results:**

The measurement uncertainty was less than four pixels for most parameters. Fardon’s classification and middle height of the affected disc were the only clinically relevant parameters statistically proven to be predictors of clinical symptoms resolution (*p* < 0.01). By combining these appropriately, we are able to identify a group of patients who have up to 22.5 times greater chance of spontaneous regression compared to others (odds ratio = 22.5, *p* < 0.001).

**Conclusion:**

By a suitable combination of the two parameters, we can select patients who are suitable candidates for conservative treatment of lumbar disc herniation and unlikely to require surgery.

## 1. Introduction

Degenerative spine disease is one of the most common pathological conditions in the population today. This condition can be accompanied by damage to the intervertebral disc and its displacement toward the spinal canal or neuroforamen, causing vertebrogenic algic syndromes, radiculopathy, and functional limitations.

Magnetic resonance imaging (MRI) is the most common method for imaging the lumbar spine in cases of these clinical symptoms, as it has high sensitivity and can best image the relationship between the disc and nerve structures without radiation burden.

The treatment of a prolapsed disc according to clinical recommendations is conservative at first, with surgical treatment only in the interval after unsuccessful conservative therapy [1]. Immediate surgical treatment is pursued only in patients with severe clinical symptomatology. There is no consensus as to whether the surgical treatment should be earlier than 4, 6, or 8 weeks after diagnosis.

Although disc protrusion may not be an irreversible condition, some patients do experience spontaneous regression of the finding that includes reabsorption or resorption of the disc and disappearance of clinical symptoms.

In the literature, one can find publications attempting to identify characteristic features that predict possible herniation resorption. The signal intensity of the herniated part of the disc is a well‐known fact. There are also papers evaluating the absolute or relative size of the herniation and its location, the Fardon’s classification, the Pfirman’s classification together with the shape of the vertebral bodies, the height of the vertebral body, Modic’s changes, and a number of other factors, including the presence of postcontrast saturation around the herniation. Thus, various factors can be monitored on MRI and a treatment strategy can be chosen according to their presence or absence [2–5].

In this work, the authors endeavored to find other predictors burdened with minimal subjective perception but that can be expressed metrically (e.g., in millimeters) rather than relatively (e.g., hyper‐ or hypo‐intensity).

## 2. Materials and Methods

This prospective single‐center project was approved by the University Hospital Brno Ethics Committee.

### 2.1. Inclusion and Exclusion Criteria

To be included into the study, a patient had to meet the following conditions: age over 18 years, signed informed consent, completed MRI, and standardized neurological examinations of the lumbar spine twice in an interval of 30–90 days without significant artifacts on MR images, disc herniation in the lumbosacral region, presentation to the neurological department with clinical pain (back or lower limb, including radiculopathy, root irritation), fulfillment of the prerequisites for possible disc surgery (herniotomy), no presence of metallic materials in the lumbar region, and no other MRI contraindications. If there was suspicion of an alternative etiology of the symptoms, it was excluded or confirmed by appropriate supplementary examinations (e.g., venous ultrasound of the lower extremities, etc.), and if confirmed, the patient was not included in the study.

### 2.2. Study Group

In this project, we examined 252 patients who were indicated for surgery and the patients consented to MRI and conservative treatment (which was the same for all patients without any changes). This conservative treatment was guided according to the general recommendations [6] and primarily focused on strengthening muscle power, stabilizing posture, increasing awareness of proper execution of daily activities, and maintaining cardiovascular fitness. When the conservative treatment did not work the second MRI and surgery were done as soon as possible. If there was a significant clinical improvement (driven by radicular symptoms), surgery was canceled, and the patient was sent for a second MRI examination. We excluded 9 patients because of planned multiple‐level operation. Another 70, resp. 48, patients were excluded because the second MRI examination was earlier, resp. later, than 30, resp. 90, days. In the first excluded group, there was only one subject with spontaneous improvement of the clinical condition, while in the second group, there were 7 subjects. The inclusion criteria were met by 125 subjects (61 women) with mean (SD) age 47.23 (11.93) years in a time window of 1‐1/2 years. In 22 subjects, there was spontaneous improvement of the clinical (neurological) condition without any intervention. The study group is described in more detail in Table 1 within the Results section.

**Table 1:** Descriptive statistics of the study population.

Parameter	Improvement^b^ *N* = 22	No improvement *N* = 103	Difference (95% CI)	*p*‐value
Sex (M)	12 (55%)	52 (50%)	4.1% (−22%–30%)	0.729^c^
Mean age (SD)	45.73 (10.97)	47.57 (12.28)	−1.8 (−7.2–3.5)	0.488^d^
Level				0.345^c^
Th12/L1^a^	0 (0%)	2 (1.9%)	−1.9% (−6.5%–2.7%)	
L1/L2^a^	0 (0%)	1 (1.0%)	−1.0% (−3.8%–1.9%)	
L2/L3^a^	1 (4.5%)	0 (0%)	4.5% (−6.9%–16%)	
L3/L4	3 (14%)	9 (8.7%)	4.9% (−13%–23%)	
L4/L5	7 (32%)	44 (43%)	−11% (−35%–14%)	
L5/S1	11 (50%)	47 (46%)	4.4% (−21%–30%)	
Pfirman’s				0.192^c^
2^a^	0 (0%)	6 (5.8%)	−5.8% (−13%–1.5%)	
3	6 (27%)	47 (46%)	−18% (−42%–5.3%)	
4	14 (64%)	42 (41%)	23% (−2.1%–48%)	
5	2 (9.1%)	8 (7.8%)	1.3% (−13%–16%)	
Modic’s				0.756^c^
0	13 (59%)	53 (51%)	7.6% (−18%–33%)	
1	3 (14%)	13 (13%)	1.0% (−16%–18%)	
2	6 (27%)	37 (36%)	−8.6% (−32%–15%)	
Fardon’s				0.090^c^
Extrusion	13 (59%)	35 (34%)	25% (−0.14%–50%)	
Foc. Protrusion	6 (27%)	52 (50%)	−23% (−47%–0.51%)	
Protrusion	1 (4.5%)	10 (9.7%)	−5.2% (−18%–8.0%)	
Sequestration	2 (9.1%)	6 (5.8%)	3.3% (−12%–19%)	
Geometry (mm/mm^2^)				
Anterior high	9.38 (2.49)	9.12 (3.16)	0.26 (−1.0–1.5)	0.676^d^
Middle high	9.67 (2.28)	8.42 (2.44)	1.2 (0.14–2.4)	0.029^d^
Posterior high	5.88 (1.64)	5.64 (1.78)	0.24 (−0.56–1.0)	0.544^d^
Disc length	34.31 (3.68)	34.59 (3.89)	−0.28 (−2.1–1.5)	0.753^d^
Herniation area	83.68 (31.14)	95.34 (52.55)	−12 (−29–5.2)	0.172^d^
Herniation depth	7.85 (2.91)	7.01 (2.35)	0.84 (−0.52–2.2)	0.218^d^
Herniation length	15.58 (5.45)	16.20 (10.15)	−0.62 (−3.7–2.4)	0.687^d^

^a^For statistical purposes, these parameters were combined into a common “other” category or were not considered due–small numbers.

^b^Disappearance of clinical symptoms without any intervention.

^c^Chi‐squared test or Fisher’s exact test.

^d^ *t*‐test.

### 2.3. Measurement

The MRI scan was performed on a Philips Ingenia 3T machine using a spinal coil. Parameters of the individual sequences are shown in Table 2.

**Table 2:** Parameters of MRI protocol.

Parameters	Sag T2	Sag T1	Ax T2
FOV (mm)	250∗280	250∗280	182∗182
Acq.Vox.Size (mm)	0.8∗0.85	0.7∗1	0.6∗0.75
Recon.Vox.Size (mm)	0.55∗0.55	0.55∗0.55	0.42
Slice thickness/gap (mm)	3/0.3	3/0.3	3/0.3
TR/TE (ms)	4200/90	630/9	3200/100
TSE factor	23	3	24
NSA	1	1	2
SENSE (Dir)	1,5 (FH)	1,5 (FH)	1,5 (RL)

Abbreviations: Acq.Vox.Size = acquisition voxel size; Dir = direction; FH = feet–head; FOV = field of view; NSA = number of signal averages; Recon.Vox.Size = reconstruction voxel size; RL = right–left; SENSE = sensitivity encoding; TE = echo time; TR = repetition time; TSE = turbo spin echo.

All measurements and quantifications were performed based on the initial (first) MRI examination. The second MRI examination (30–90 days after the first) was used to check visually for hernia resorption. Both MRI examinations were complemented by neurological examination in the clinic (not in the emergency room). The main decision criterion for group division is not visual resorption of the hernia, however, but spontaneous improvement of the patient’s clinical (neurological) condition without any intervention.

Internationally accepted methods were used for characterizing the MRI examination according to the recommended procedures. Pfirman’s classification was used to determine disc quality changes [7], Fardon’s classification to assess disc herniation [8], and Modic’s classification for vertebral‐end plate changes [9].

**Figure 1:**
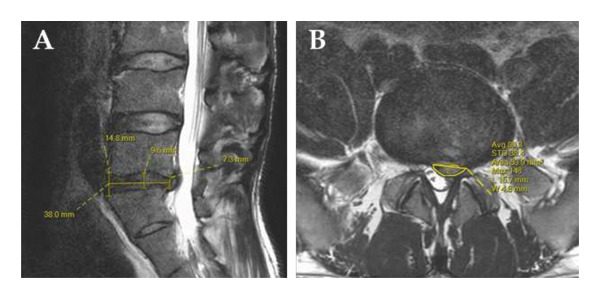
Demonstration of disc and herniation measurements on sagittal (A) and transverse (B) T2‐w images.

Sagittal T2‐w images were used to measure disc length (from anterior to posterior edge of the junction of two adjacent vertebral bodies) and to measure disc height in three regions: the anterior and posterior disc height at the junction of the anterior and posterior edges of the vertebral bodies, respectively, and the middle disc height (MDH) at the midpoint of the disc length (Figure 1(A)).

Based on the transverse T2‐w images, the length of the herniation, the greatest distance of the herniation from its base (depth), and the area of the herniation in this layer were measured (Figure 1(B)).

Measurements of the above parameters were performed by two radiologists (TH, MS) with different degrees of experience and lengths of practice (5 and 2 years, respectively) and one medical student (AČ), all in blinded regime. All of them first received training on how to perform the measurements and assessments and were provided with an accurate manual prepared by a radiologist with 25 years of experience and certification in neuroradiology (AŠ‐P). That radiologist also conducted the training and subsequently consulted on any questionable situations. Intra‐ and inter‐reproducibility were determined based on repeated measurements by one evaluator (AČ) on 11 (9.1%) patients and by two evaluators (TH, MS) on 26 (20.8%) patients.

### 2.4. Statistics

Descriptive statistics comparing groups of patients with and without clinical improvement were statistically evaluated using *X* ^2^ test and Student′s *t*‐test.

Inter‐ and intra‐reproducibility were verified statistically using Cronbach’s alpha according to the average of absolute and relative differences based on the following formulas:

(1) Abs.diff.=Measure1−Measure2

(2) Rel. diff.=Measure1−Measure2Measure1+Measure2/2 

The degree of influence of each of the studied parameters on improvement of the patient’s condition was statistically evaluated using univariate linear regression.

Receiver operating characteristic (ROC) analysis was used to determine the optimal cutoff value of the parameter MDH for each spinal level in relation to improvement in the patient’s condition using the method of maximizing the sum of specificity and sensitivity. By applying the obtained cutoff values to the parameter MDH, the set was divided according to fulfillment or nonfulfillment of the cutoff criteria. We called this new binary variable “binary middle disc height” or BMDH.

The joint effect of the statistically and clinically significant parameters (BMDH and Fardon’s classification) on improvement of the patient’s condition was statistically evaluated by multivariable logistic regression.

An alpha significance level of 0.05 was used for all statistical tests, which were made using the statistical softwares SPSS [10] and R [11].

## 3. Results

Descriptive statistics of the cohort and statistical comparison of patients with and without clinical symptoms disappearance without any intervention are presented in Table 1. According to the results, it can be seen that, based on those descriptive parameters used, there is no statistically significant difference between the groups with and without clinical symptoms disappearance.

Inter‐ and intra‐reproducibility assessments are shown in Table 3. The mean absolute differences are on the order of units of millimeters, representing relative differences on the order of tens of percent. Although the relative deviations reach high numbers, they are lower voxel counts, except that in the herniation area, the number of voxels increases quadratically with the error. The length and middle height of the disk appear to be the most reproducible in relative terms. The least reproducible parameter is the herniation area.

**Table 3:** Reproducibility of individual parameter measurements in terms of intra‐ and inter‐repeatability.

Parameter		Cronbach’s alpha		Mean Abs. Dif. [mm^a^ (vox)]		Mean Rel. Dif. (%)	
		Intra	Inter	Intra	Inter	Intra	Inter
Pfirman’s		1.00	0.81	—	—	—	—
Modic’s		1.00	0.94	—	—	—	—
Fardon’s		0.91	0.60	—	—	—	—
Disc	Anter.	0.57	0.80	1.5 (2.7)	2.1 (3.8)	21.3	25.3
	Middle	0.81	0.61	0.8 (1.5)	2.1 (3.8)	14.3	27.8
	Poster.	0.21	0.75	1.1 (2.0)	1.6 (3.0)	26.6	34.6
	Length	0.95	0.88	2.0 (3.6)	2.4 (4.3)	5.7	6.7
Herni.	Area	0.12	0.71	51.1 (169)	34.7 (115)	54.7	41.5
	Depth	0.19	0.68	2.0 (3.6)	2.0 (3.7)	29.5	27.5
	Length	0.77	0.17	4.3 (7.8)	7.9 (14.3)	26.6	60.2

Abbreviations: Abs. = absolute; Anter. = anterior; Dif. = difference; Herni. = herniation; Poster. = posterior; Rel. = relative; vox = voxel count.

^a^Herniation area in mm^2^.

The partial results of the multivariable logistic regression when omitting or merging groups with small number of subjects are shown in Table 4. Four observed parameters showed statistical significance. If a patient has a disability classified as extrusion based on the Fardon’s classification, the odds ratio (OR) is 8.95 (*p* = 0.009), which means that the odds of improving the patient’s health status without the need for surgery are 8.95 times those compared to when the classification is focal protrusion. We can see that the 95% CI OR is quite narrow, indicating this parameter to be robust. The second significant parameter was MDH, in which case the OR was 3.12 (*p* < 0.001), meaning that patients with a larger MDH were more likely to improve their health status without the need for surgery. When level of herniation was classified as other, effect of level was proven to be statistically significant, but the 95% CI OR is wide and only four subjects were in this category. For the same reasons, we consider level of herniation as well as the last statistically significant parameter (Pfirman’s) to be unreliable, and for further statistical analysis, we used only Fardon’s classification and MDH.

**Table 4:** Results of univariable logistic regression for the observed parameters.

Parameter	N	OR (95% CI)	*p*‐value
Sex			
Men	64	Ref.	
Female	61	0.40 (0.06–2.41)	0.32
Age	125	0.73 (0.90–1.03)	0.35
Level			
L3/4	12	Ref.	
L4/5	51	0.58 (0.06–5.84)	0.63
L5/S1	58	1.88 (0.20–20.3)	0.59
Other	4	291 (4.11–25,855)	**0.008**
Modic’s			
0	66	Ref.	
1	16	2.51 (0.21–24.4)	0.44
2	43	1.15 (0.26–5.05)	0.85
Pfirman’s			
3	53	Ref.	
4	56	3.91 (1.01–17.5)	0.057
5	10	20.2 (1.25–370)	**0.033**
Fardon’s			
Focal protrusion	59	Ref.	
Extrusion	48	8.95 (1.91–52.8)	**0.009**
Protrusion	11	0.70 (0.02–8.46)	0.80
Sequestration	8	5.81 (0.46–65.0)	0.15
Disc height			
Anterior	125	0.75 (0.51–1.04)	0.11
Middle	125	3.12 (1.83–6.19)	**< 0.001**
Posterior	125	0.79 (0.41–1.32)	0.38
Disc length	125	0.83 (0.63–1.09)	0.19
Herniation			
Area	125	0.99 (0.97–1.01)	0.19
Depth	125	1.15 (0.84–1.73)	0.42

*Note:* Spontaneous hernia resorption without any intervention was used as the dependent variable. Bold values represent statistical significance.

Abbreviations: CI = confidence interval; N = number of subjects in the group; OR = odds ratio; Ref. = reference.

While the Fardon’s classification is independent of the level of disability, the MDH is level dependent [12]. Therefore, using ROC analysis, cutoff values for the variable MDH were determined for the three levels occurring most frequently (Table 5). Based on meeting or not meeting the cutoff values, patients were divided into two groups (i.e., according to BMDH). The number of subjects with greater or lesser MDH is shown in Table 5.

**Table 5:** Group distribution based on middle disc height according to not meeting/meeting cutoff value.

	L3/4	L4/5	L5/S1
ROC			
Cutoff (mm)	9.4	8.9	9.1
Sens./spec.	0.65/0.56	1.0/0.52	0.81/0.68
Number of subjects with middle disc height greater or lower than the cutoff			
Lower	6	23	34
Greater	6	28	23

Abbreviations: ROC = receiver operating characteristic; Sens. = sensitivity; Spec. = specificity.

**Figure 2:**
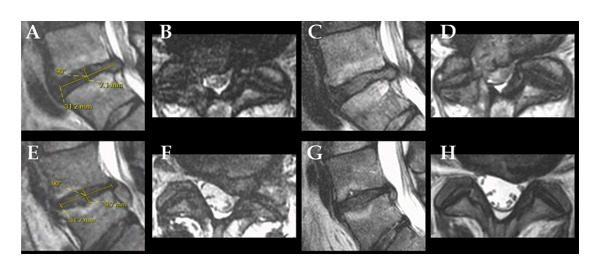
Demonstration of disk measurements. Example of patient without clinical improvement without any intervention with extrusion and middle disc height below the threshold (upper row) in sagittal plane (A, C) and axial plane (B, D) of the first MRI examination (A, B) and the second (C, D). Example of patient with clinical improvement without any intervention with extrusion and middle disc height above the threshold (lower row) in sagittal plane (E, G) and axial plane (F, H) of the first MRI examination (E, F) and the second (G, H).

Multivariable linear regression of the interaction between Fardon’s classification and BMDH showed statistical significance of the two parameters (Table 6). If the MDH is higher than the established cutoff value and the Fardon’s disc classification is also extrusion, then the patient has high odds (OR 22.5, *p* < 0.001) for improvement in health status without the need for surgery compared to patients with MDH values lower than the established cutoff. The example of two patients is in Figure 2. The lower bound of the 95% CI OR is 5.51, which is a high value, and it could be interpreted as indicating strong robustness of this predictor. Similarly, even with a higher MDH and focal protrusion, there is a realistic chance of spontaneous improvement without any intervention in patient health status without the need for surgery (OR = 4.62, *p* = 0.04) compared to patients with MDH lower than the established cutoff. The results of the other combinations cannot be considered statistically significant.

**Table 6:** Results of multivariable logistic regression of Fardon’s classification interaction with binary middle disc height (BMDH).

Parameter	N	Event N	OR (95% CI)	*p*‐value
BMDH and extrusion	47	12	22.5 (5.51–119)	**< 0.001**
BMDH and focal protrusion	55	6	4.62 (1.13–23.2)	**0.040**
BMDH and protrusion	11	1	3.33 (0.15–31.3)	0.33
BMDH and sequestration	8	2	> 10^9^ (0.0–Inf.)	> 0.99

*Note:* Bold values represent statistical significance.

Abbreviations: Event N = number of cases with clinical improvement; CI = confidence interval; Inf. = infinity; N = number of cases in the group; OR = odds ratio.

## 4. Discussion

In some patients with lumbar disc herniation, the clinical symptoms may resolve spontaneously (without any intervention) and therefore surgery is not necessary. Various time intervals between initial examination and spontaneous resolution of symptoms are reported in the literature [13, 14]. If the probability for spontaneous resolution of symptoms could be determined at the initial examination, this could lead to a more accurate and timely clinical assessment with sufficient time for conservative treatment. The question remains, however, what are the predictors of spontaneous resolution of symptoms?

A frequently reported predictor is the volume of the herniated disc [2], but this remains ambiguous because there are studies that, on the other hand, have not shown size of the herniated disc to be a reliable predictor [15]. Therefore, we did not address herniation volume in our study. To some extent, this ambiguity may be due to the necessity of acquiring a 3D sequence with thin slice thickness, which is not part of a standard MRI protocol, and the determination of herniation volume from 2D images with thicker slice thickness may be inaccurate and not reproducible.

The composition of the herniated portion of the disc, specifically a higher proportion of nucleus pulposus, promotes disc resorption [16], and there is also work supporting a theory that, conversely, a larger proportion of cartilaginous material from the covering discs inhibits resorption [17] A greater proportion of cartilaginous material can be approximated to some extent by Modic’s classification [18], because a presence of Modic’s changes may have an effect on eventual disc reabsorption. That was not demonstrated in our study, however. In a review study drawing upon 69 articles, Viswanathan et al. came to the same conclusion, writing “*There is no conclusive evidence on the causative role of MC (Modic changes) in chronic low back pain (LBP) or any influence on the long term outcome in patients with LBP or lumbar disc herniations (LDH).”* [19]

As early as the 1980s, Doita et al. had demonstrated that macrophage infiltration of herniated discs corresponds to a greater likelihood of resorption [20]. Almost 40 years later, the work of Djuric et al. demonstrated that type of disc herniation is significantly associated with degree of macrophage infiltration [21], with disc extrusions having greater macrophage infiltration than protrusions. Taken in conjunction with the results of Doita et al., this implies that disc extrusions have a greater chance of herniation resorption. We reached the same conclusion in our work, where the Fardon’s classification was shown to be a statistically significant predictor of symptom resolution [8].

Our study showed that the probability of resorption increases with middle height of the herniated disc. One theory to explain this result is that the internal structure of the affected disc is not yet degraded enough to allow the spine to collapse. The paraspinal muscles and overall stabilizing system also play a significant role, and this may help to maintain the height of the disc and thus create a better environment for its resorption. If the disc height is reduced, the distribution of forces and pressure on the disc and thus on the herniated part may change, and this may result in a worsening of the clinical condition (e.g., due to increased nerve root compression). This theory can be supported by the work of Kjaer et al., who longitudinally followed the development of herniation in 106 patients at three time points, namely at 41, 45, and 49 years of life [22]. The authors concluded that “*larger herniation size predicted a reduction in both dural sac area and disc height over a four- to eight-year period.*” This also could be interpreted to mean that a decrease in disc height predicts an increase in herniation and thus probably a clinical deterioration of the patient.

Based on the previously stated considerations and the results of univariable analysis, it is logical to use Fardon’s classification and MDH for multivariable logistic regression, which shows that combination of these two predictors greatly increases the odds of predicting spontaneous resolution of clinical problems and thus the successful use of conservative treatment in such patients.

Analysis of intra‐ and inter‐reproducibility of parameter measurements showed that some are more reproducible than others. Specifically, reproducibility of herniation depth and area which is one of the reasons why we did not measure volume of herniation, and it confirms the well‐known fact that it is difficult to define the boundaries of hernia and disc. Intra‐reproducibility of Fardon’s classification is substantial, and inter‐reproducibility is moderate; however, reproducibility was verified by specialists with varying experience and therefore achieved lower values than in studies conducted by similarly experienced assessors [23]. In particular, the relative deviations look very imprecise, reaching up to several tens of percent. It should be noted, however, that with a deviation of 2.1 mm and a pixel size of the standard sequence of 0.55 mm, this error represents only 3.8 pixels, and this can be seen as a relatively small measurement error in the result. However, this shortcoming can be compensated by emerging machine learning methods, where, for example, Spine Explorer or CoLumbo seem to be ideal tools for quantifying the lumbar spine with high accuracy [24, 25].

Another weakness of the project is the use of just one magnetic resonance system, which may be limiting for determination of the boundary height of the discs used to establish the binary variable BMHD as one of the input parameters to the multivariable logistic regression model. When the images of other sequence setting or MR vendor are used, the resolution or contrast differences might lead into other cutoff values. This should be the subject of further multicentric research.

The limited follow‐up window (30—90 days) may also be a limitation of the work. However, this interval was chosen to avoid external influences that may affect the improvement of clinical symptoms during long‐term follow‐up, and it is questionable whether it was appropriately chosen. Setting the lower limit (30 days) is justified, because patients were in a more serious clinical state and underwent the surgery earlier. Setting an upper limit of 90 days is more difficult because conservative treatment can take even longer, but we know from our experience and other studies [26] that patients are impatient and return to full load prematurely, which we thought was a big risk, so we think the 90‐day limit is a good compromise.

In 2010, a new classification method (MSU) was introduced [27], which has experienced a resurgence in recent years. This classification has the potential to better characterize nerve compression and, consequently, to correlate more accurately with clinical symptoms of nerve irritation. Therefore, it would be advisable to include this classification in future studies addressing this issue.

In some studies, efforts have been made to objectify clinical symptoms using standardized scales (e.g., ODI, VAS, or SF‐36). However, the aim of this work is to develop and validate a methodology for routine clinical practice, where such standardized scales are not commonly employed. Since we are working with data from patients undergoing routine examinations, we do not have access to these objective assessment scales. While the use of these scales could improve standardization, we are concerned that, due to their complexity, they are unlikely to become part of routine clinical assessments in the near future.

Although the sample size might be regarded as a limitation of this study, our sample is one of the largest in comparison with those of previous work on this topic. Moreover, the upper or lower bounds of the 95% CI OR for clinically significant predictors are quite far from 1 and narrow, indicating that robustness of these predictors can be considered strong and the study sample size is sufficiently large.

## 5. Conclusion

In our study, a new predictor of disc reabsorption without any intervention was found by combining the well‐known Fardon’s classification and the measurable parameter MDH. In a cohort of patients, we were able to find the MDH threshold where it is possible to opt for conservative management and postpone surgery, thus reducing the cost of patient treatment, sparing the patient from invasive procedures associated with long recovery, and reducing the burden on surgical centers.

## Ethics Statement

This study was conducted in accordance with the principles of the Declaration of Helsinki and was approved by the University Hospital Brno Ethics Committee. All patients signed informed consent.

## Disclosure

All authors approved the final manuscript.

## Conflicts of Interest

The authors declare no conflicts of interest.

## Author Contributions

Conception and design: Dostál, Smrčka, and Šprláková‐Puková. Provision of study materials or patients: Kryštofová and Sova. Collection and assembly of data: Čellár, Habas, Kryštofová, Sova, and Straka. Data analysis and interpretation: Barusová, Dostál, Smrčka, and Šprláková‐Puková. Manuscript writing: all authors.

## Funding

This work was supported by the Ministry of Health, Czech Republic—conceptual development of research organization (FNBr, 65269705).

## Data Availability

The datasets used and analyzed during the current study are available from the corresponding author upon reasonable request.
